# Supplementary material for: Microbial Transformation of the Sesquiterpene Lactone, Vulgarin, by Aspergillus niger
Source: Molecules. 2023 Apr 26;28(9):3729. doi: 10.3390/molecules28093729 (PMC10180108; doi:10.3390/molecules28093729)
Supplement: Supplementary file 1 [file molecules-28-03729-s001.zip › molecules-2340883-supplementary.pdf]

Supporting information

Microbial Transformation of the Sesquiterpene Lactone, Vulgarin, by *Aspergillus niger*

Reem A. ElGamal[1], Amal A. Galala[1], Maged S. Abdel-Kader[2, 3], Farid A. Badria[1, *]and Amal F. Soliman[1]

[1] *Pharmacognosy Department, Faculty of Pharmacy, Mansoura University, El Mansoura 35516, Egypt*

[2] *Department of Pharmacognosy, College of Pharmacy, Prince Sattam Bin Abdulaziz University, Al-Kharj 11942, Saudi Arabia*

[3] *Department of Pharmacognosy, Faculty of Pharmacy, Alexandria University, Alexandria 21215, Egypt*

[*]Corresponding authors:

Farid A. Badria Tel.: (+20)1001762927

E-mail address: faridbadria@gmail.com, badri002@mans.edu.eg ORCID: https:// orcid.org/0000-0001-7230-3105

Co-authors E-mails:

Reem A. ElGamal: reem.a.elgamal@hotmail.com

Amal A. Galala: amal_galala@yahoo.com

Maged S. Abdel-Kader: m.youssef@psau.edu.sa

Amal F. Soliman: ORCID: https:// orcid.org/0000-0003-3320-0685

Table of Contents	Page
**Figure S1.** ^{1}H NMR spectrum of **(1)** ($CDCl_3$, 700 MHz).	3
**Figure S2.** COSY spectrum of **(1)**	4
**Figure S3.** ^{13}C-NMR spectrum of **(1)** ($CDCl_3$, 175 MHz).	5
**Figure S4.** DEPT 135 spectrum of **(1)**	6
**Figure S5.** HSQC spectrum of **(1)**	7
**Figure S6.** HMBC spectrum of **(1)**	8
**Figure S7.** FAB-MS spectrum of **(2)**	9
**Figure S8.** ^{1}H-NMR spectrum of **(2)** ($CDCl_3$, 500 MHz).	10
**Figure S9.** COSY spectrum of **(2)**	11
**Figure S10.** NOESY spectrum of **(2)**	12
**Figure S11.** ^{13}C-NMR spectrum of **(2)** ($CDCl_3$, 125 MHz).	13
**Figure S12.** HRESIMS spectrum of **(3a)**	14
**Figure S13.** ^{1}H-NMR spectrum of **(3a)** ($CDCl_3$, 500MHz).	15
**Figure S14.** COSY spectrum of **(3a)**	16
**Figure S15.** NOESY spectrum of **(3a)**	17

**Figure S16.** ^{13}C-NMR spectrum of **(3a)** ($CDCl_3$, 125 MHz).	18
**Figure S17.** HSQC spectrum of **(3a)**	19
**Figure S18.** HMBC spectrum of **(3a)**	20
**Figure S19.** HRESIMS spectrum of **(3a,b)**	21
**Figure S20.** ^{1}H-NMR spectrum of **(3a,b)** ($CDCl_3$, 500 MHz).	22
**Figure S21.** COSY spectrum of **(3a,b)**	23
**Figure S22.** ^{13}C-NMR spectrum of **(3a,b)** ($CDCl_3$, 125 MHz).	24
**Figure S23.** DEPT 135 spectrum of **(3a,b)**	25
**Figure S24.** HMBC spectrum of **(3a,b)**	26
**Figure S25.** HSQC spectrum of **(3a,b)**	27

NMR spectra of vulgarin (1):

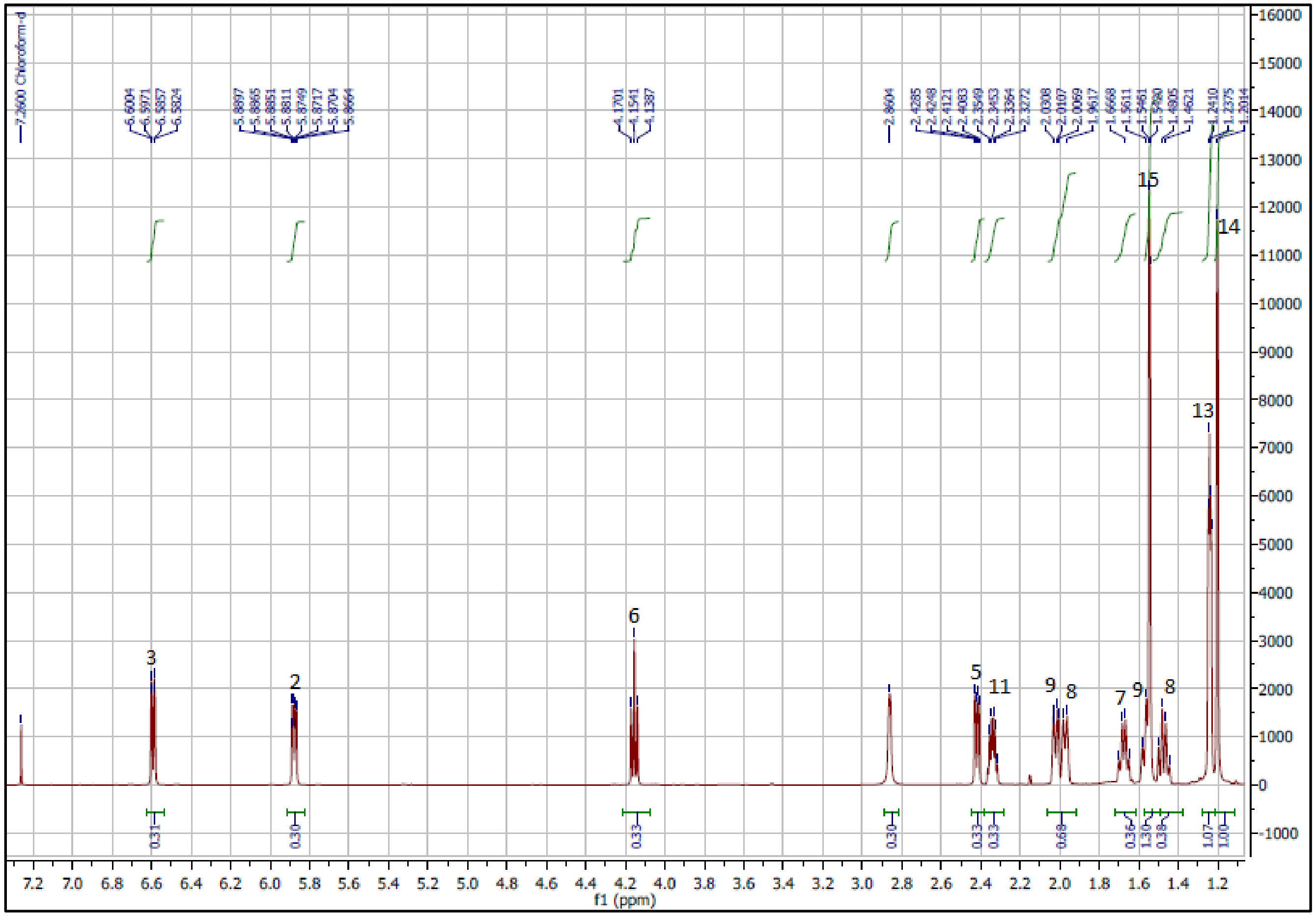

Figure S1. ^{1}H NMR spectrum of vulgarin **(1)** ($CDCl_3$, 700 MHz).

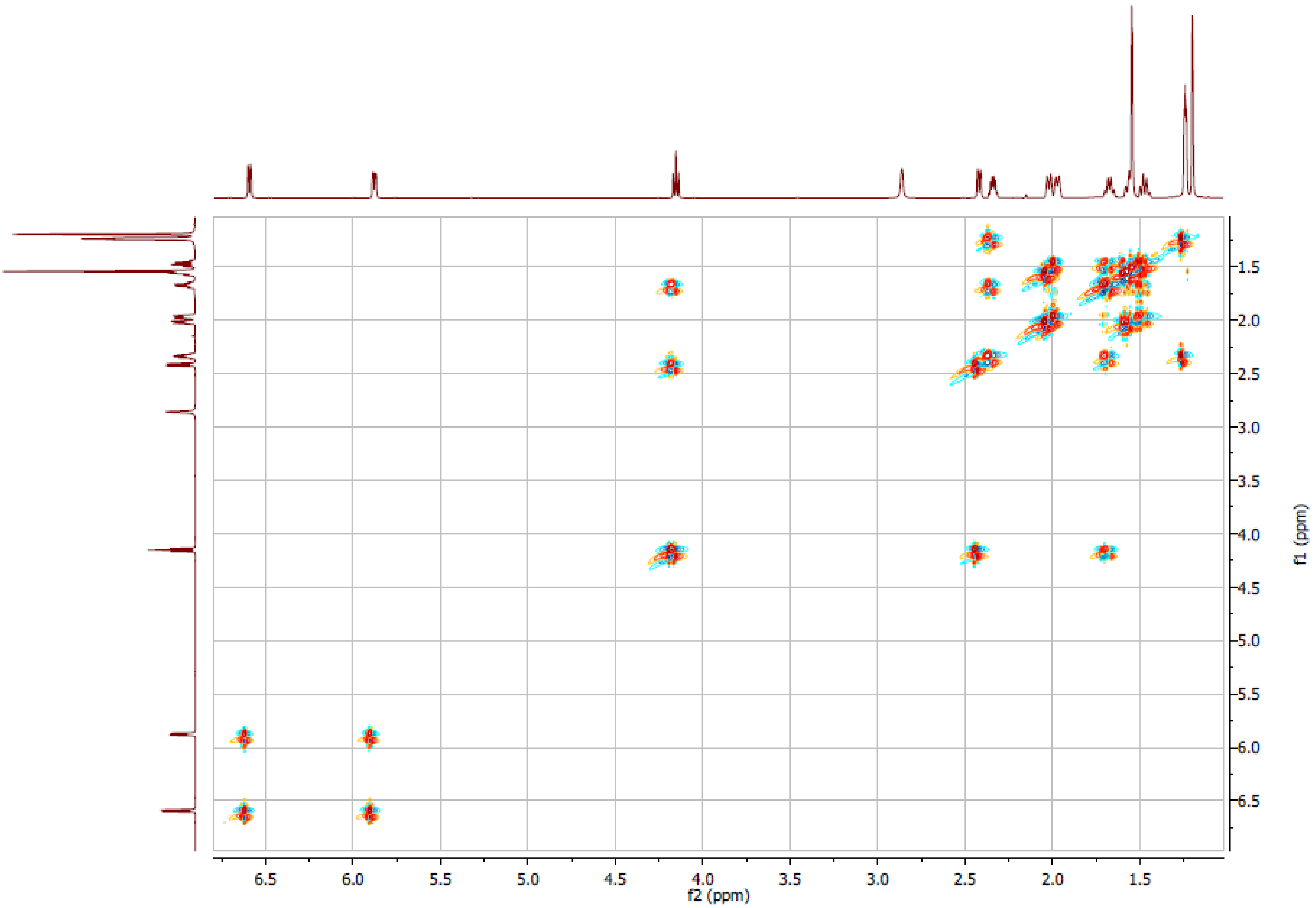

Figure S2: COSY spectrum of Vulgarin **(1)**

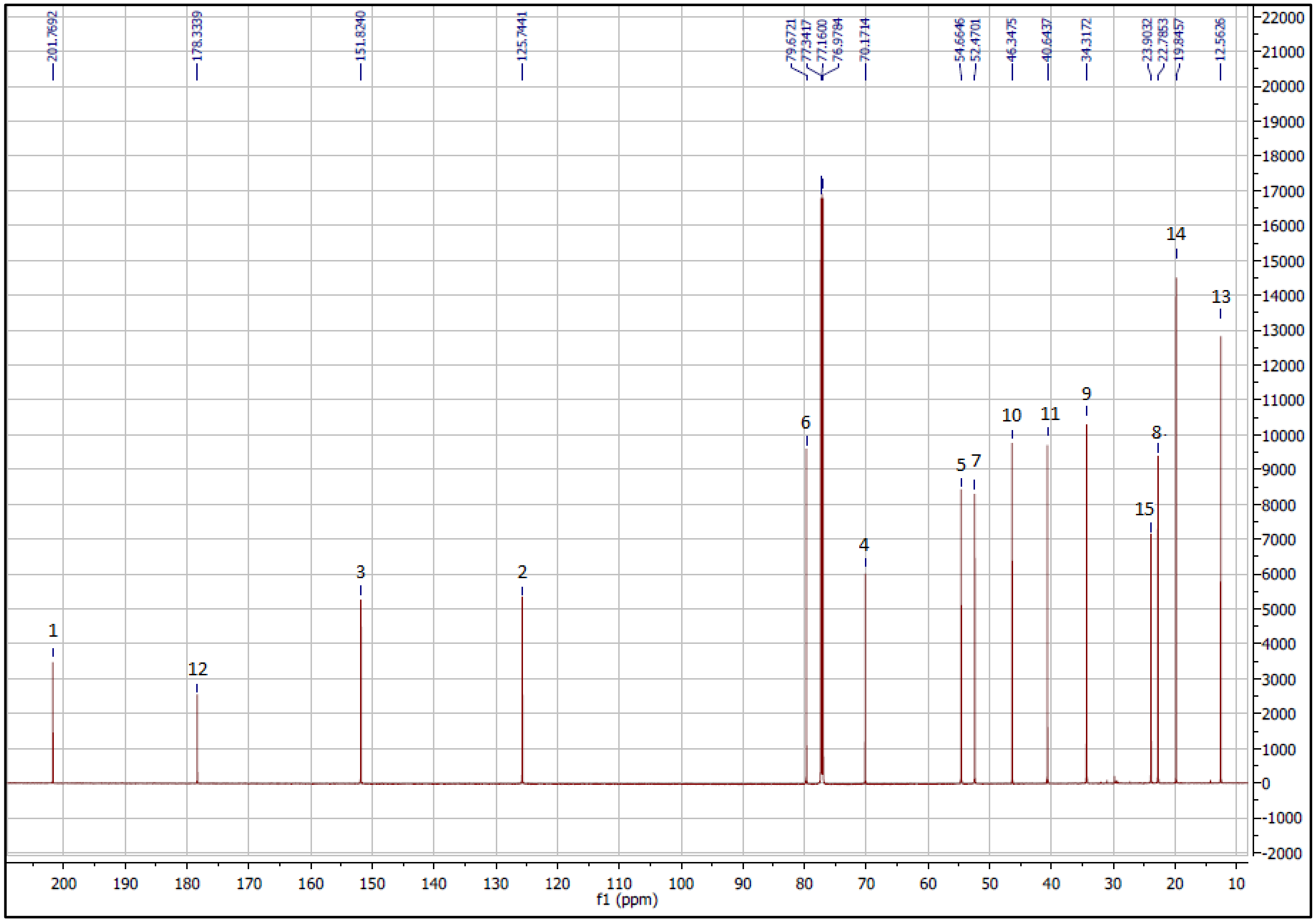

Figure S3: ^{13}C-NMR spectrum of Vulgarin **(1)** ($CDCl_3$, 175 MHz).

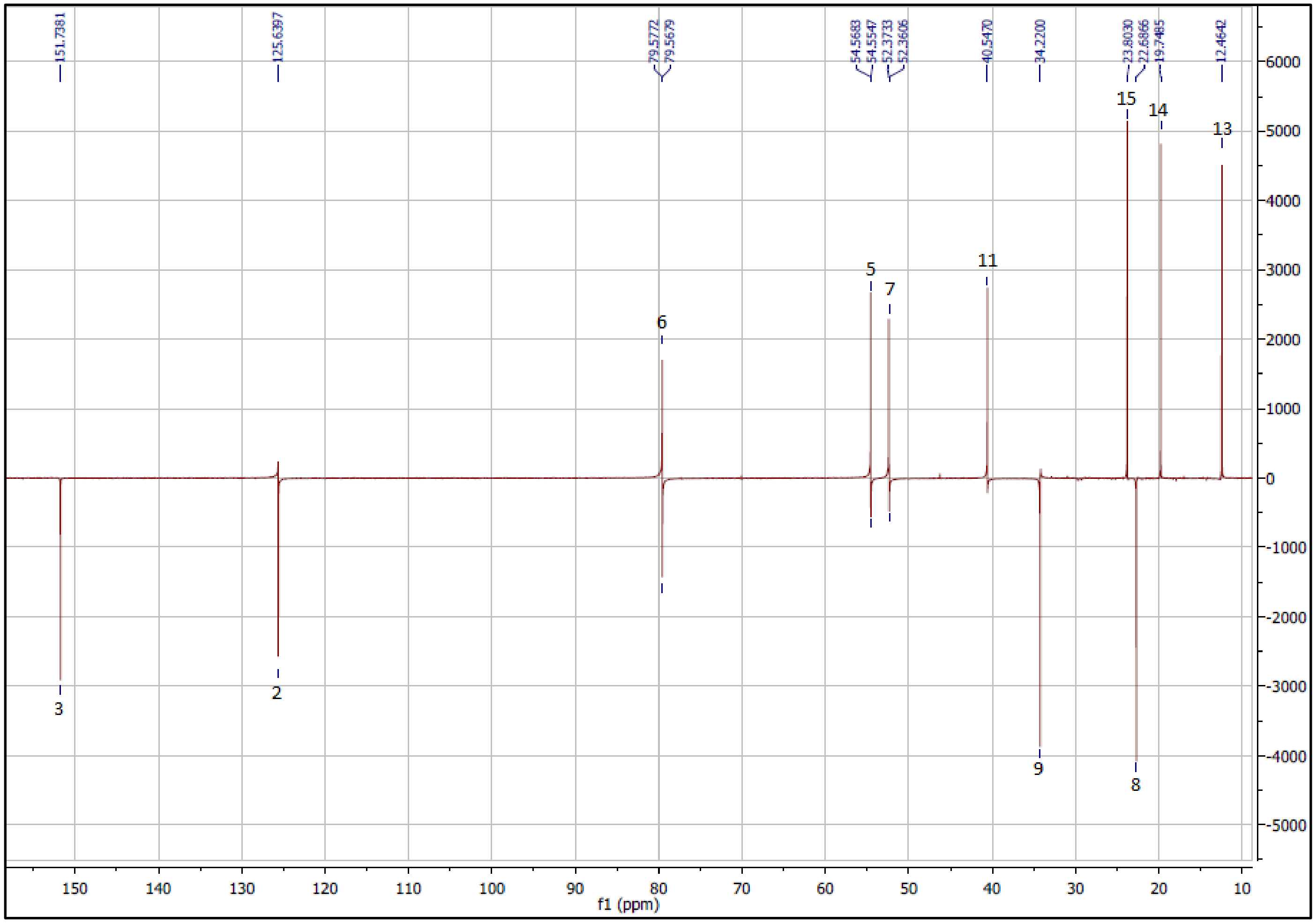

Figure S4: DEPT 135 spectrum of Vulgarin **(1)**

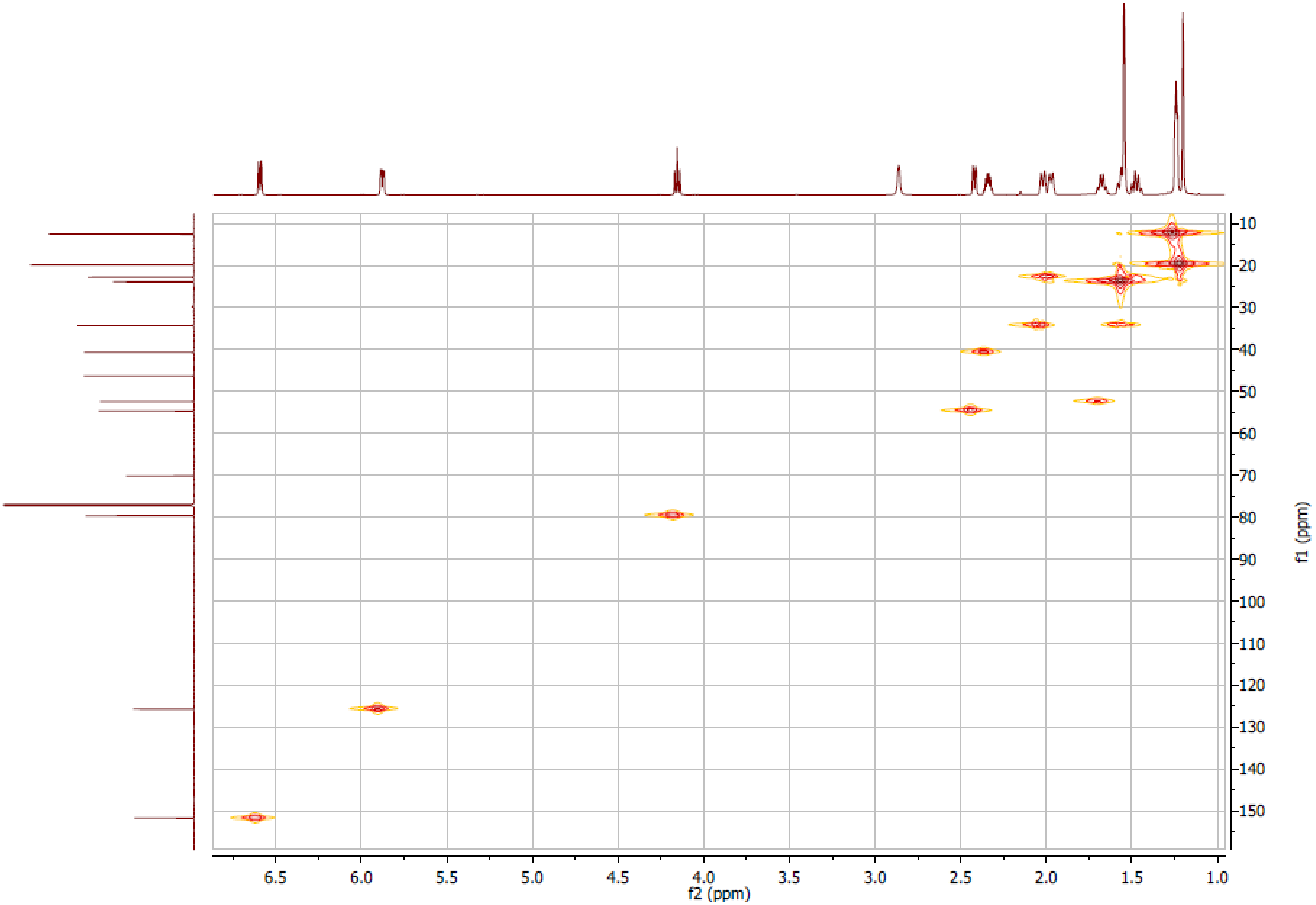

Figure S5: HSQC spectrum of Vulgarin **(1)**

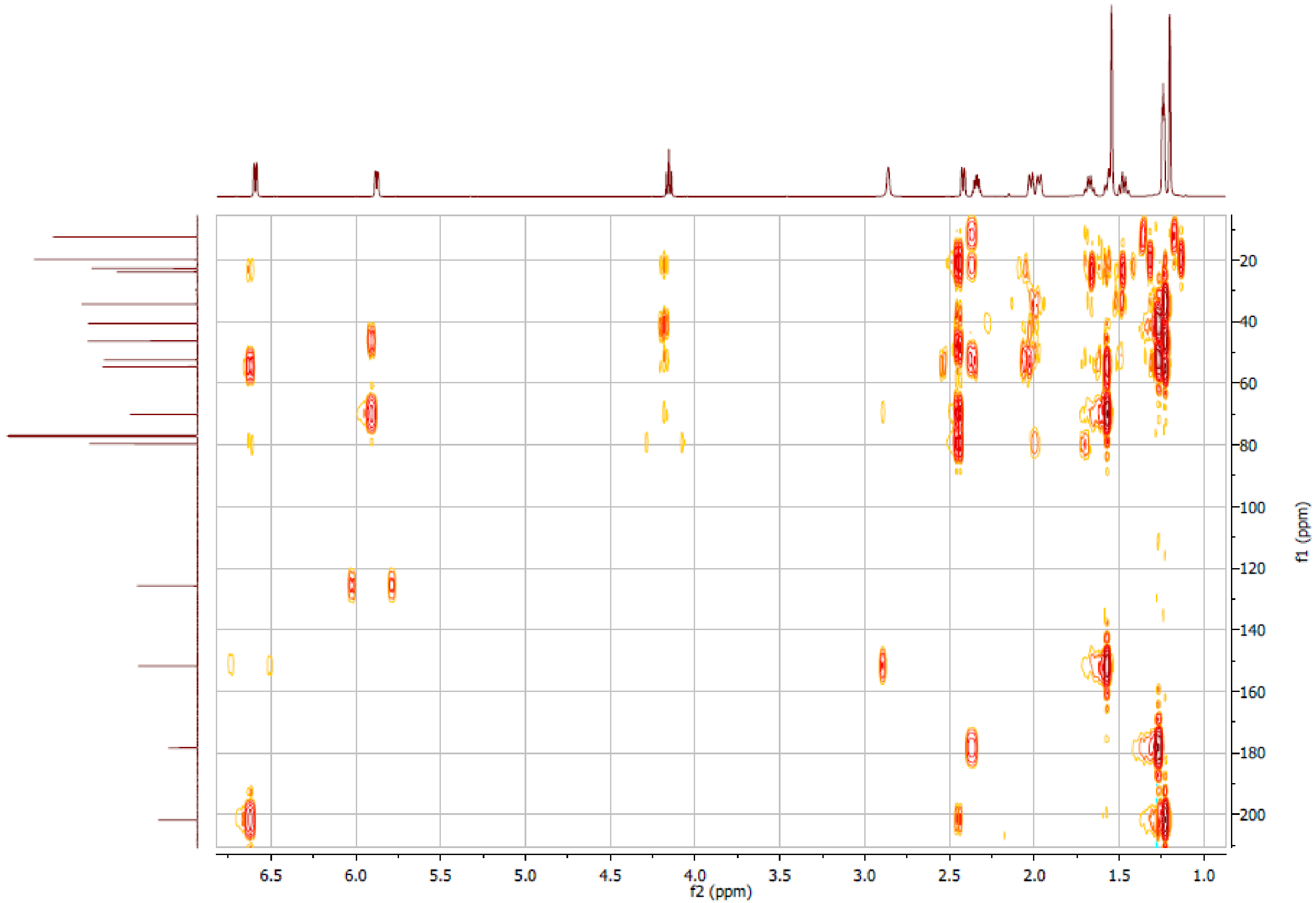

Figure S6: HMBC spectrum of Vulgarin **(1)**

NMR spectra of first metabolite (2)

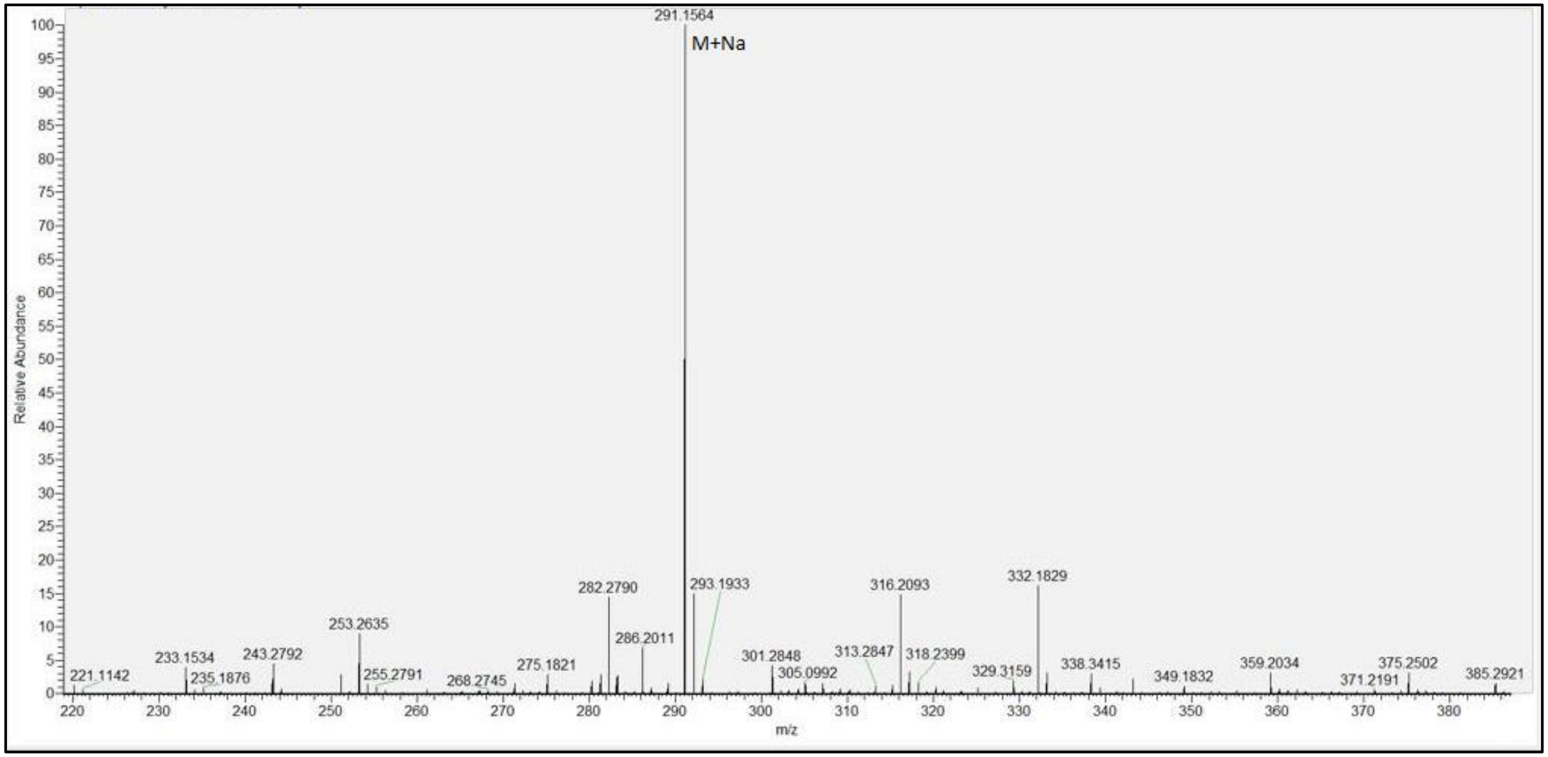

Figure S7: HRESIMS spectrum of **(2)**

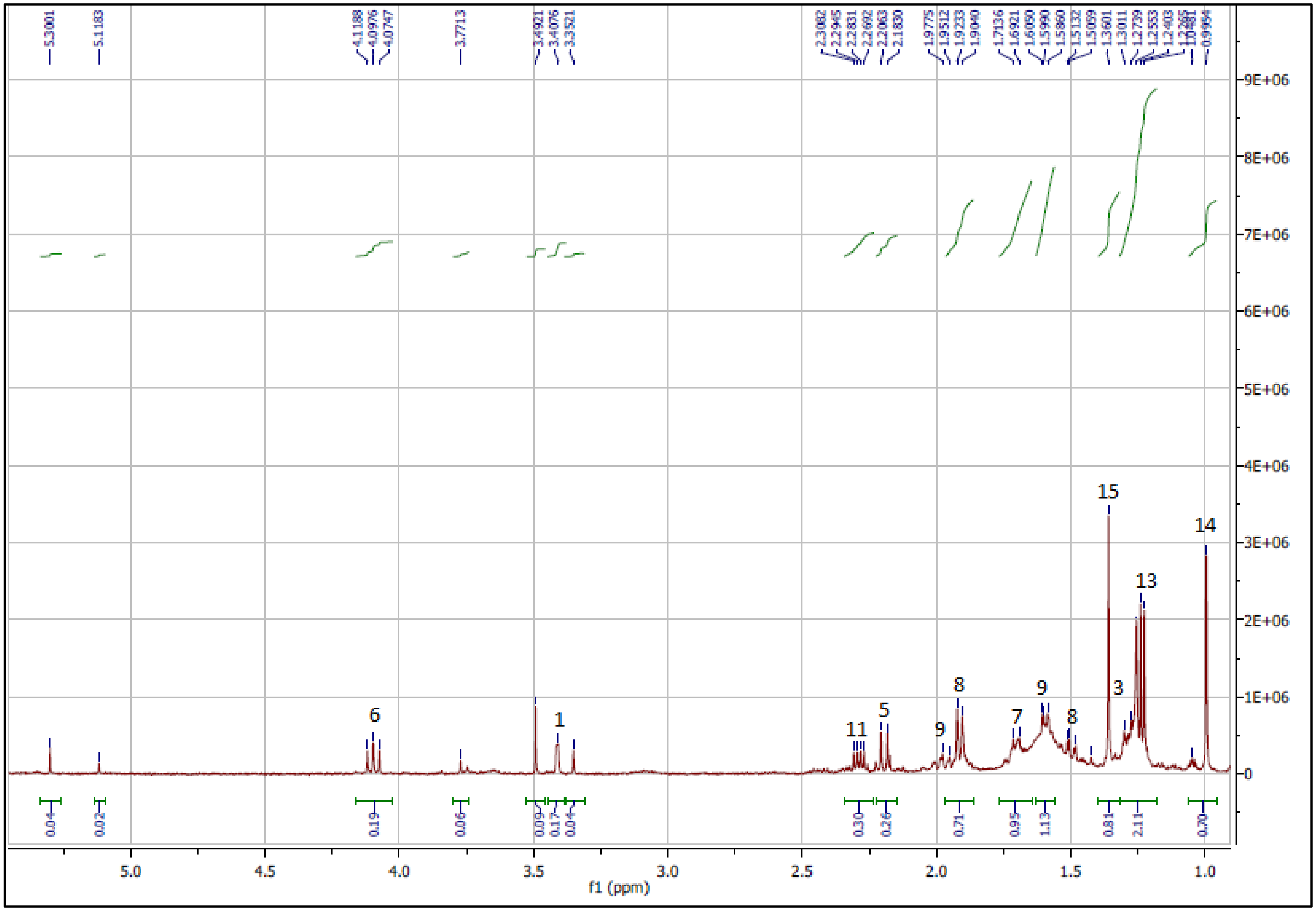

Figure S8: ^{1}H-NMR spectrum of **(2)** ($CDCl_3$, 500 MHz).

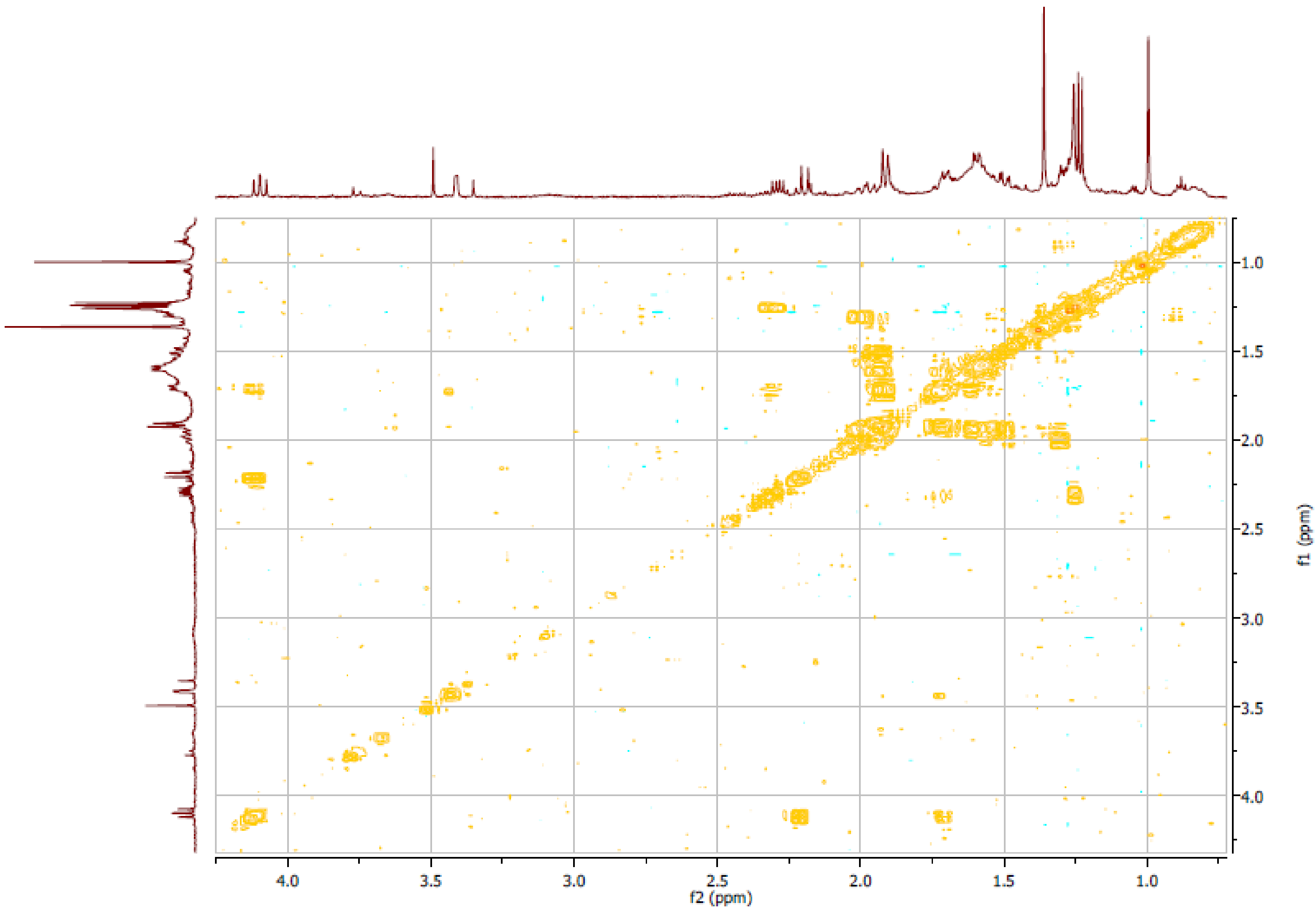

Figure S9: COSY spectrum of **(2)**

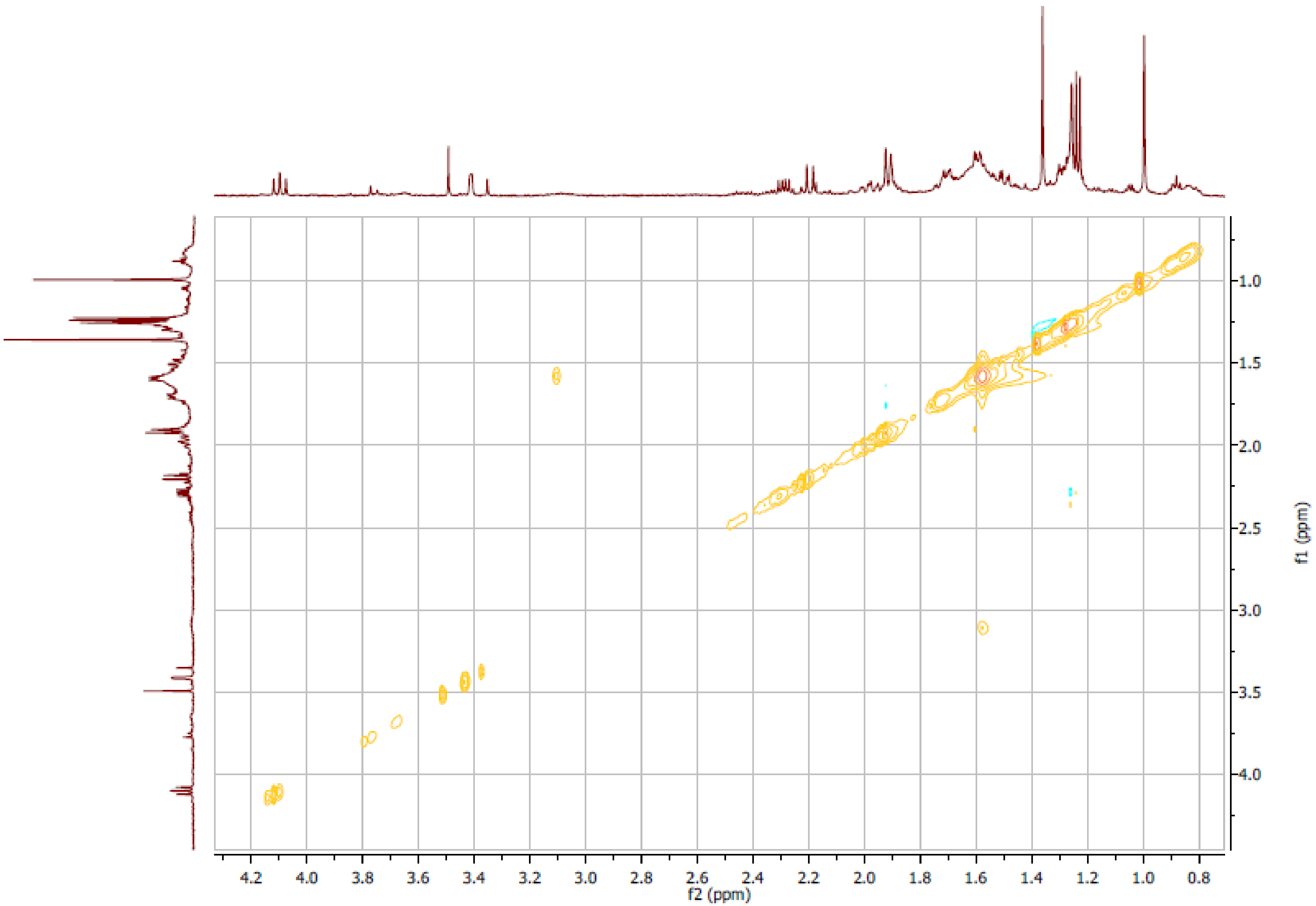

Figure 10: NOESY spectrum of **(2)**

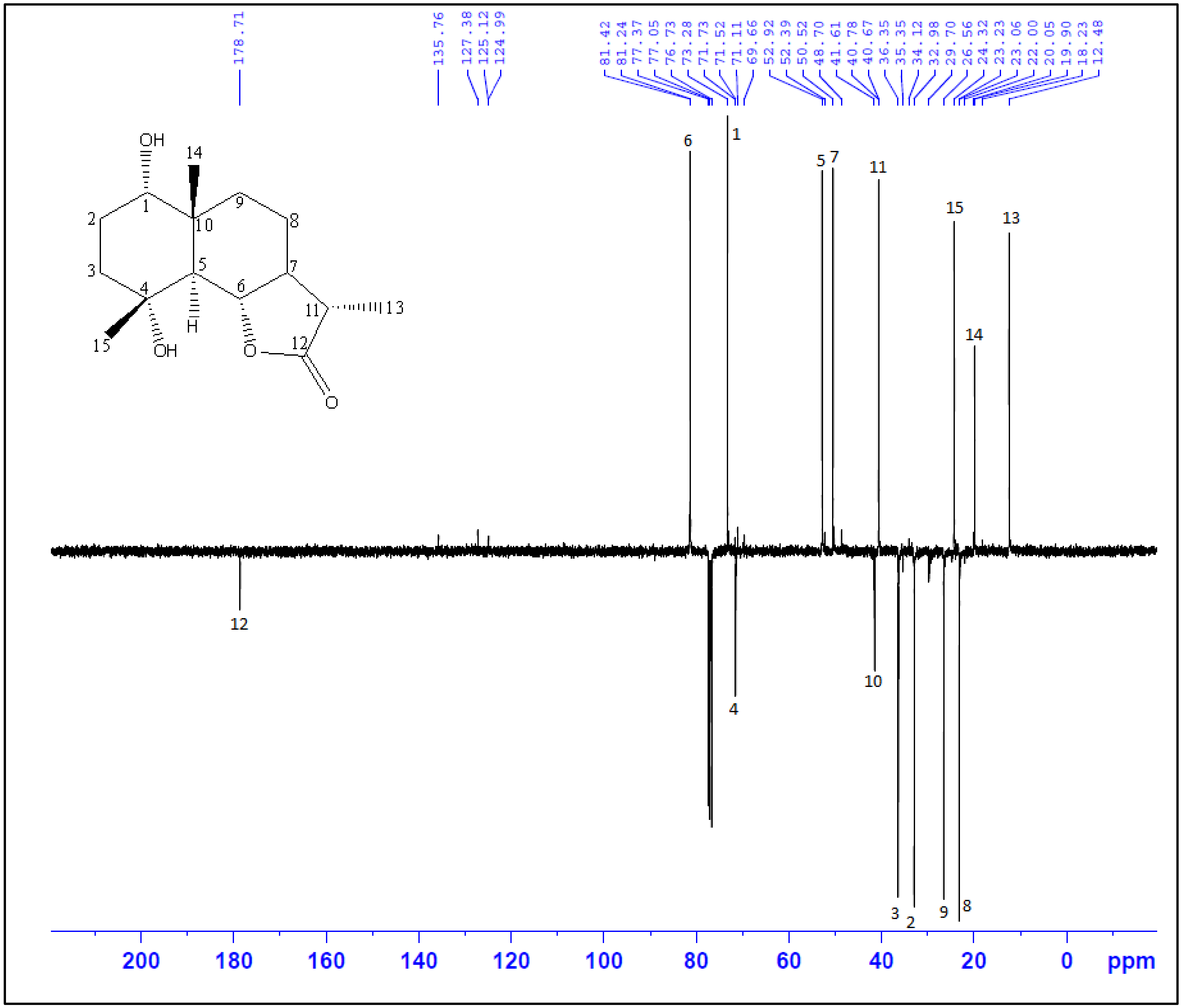

Figure S11: ^{13}C-NMR spectrum of **(2)** ($CDCl_3$, 125 MHz).

NMR spectra of second metabolite (3)

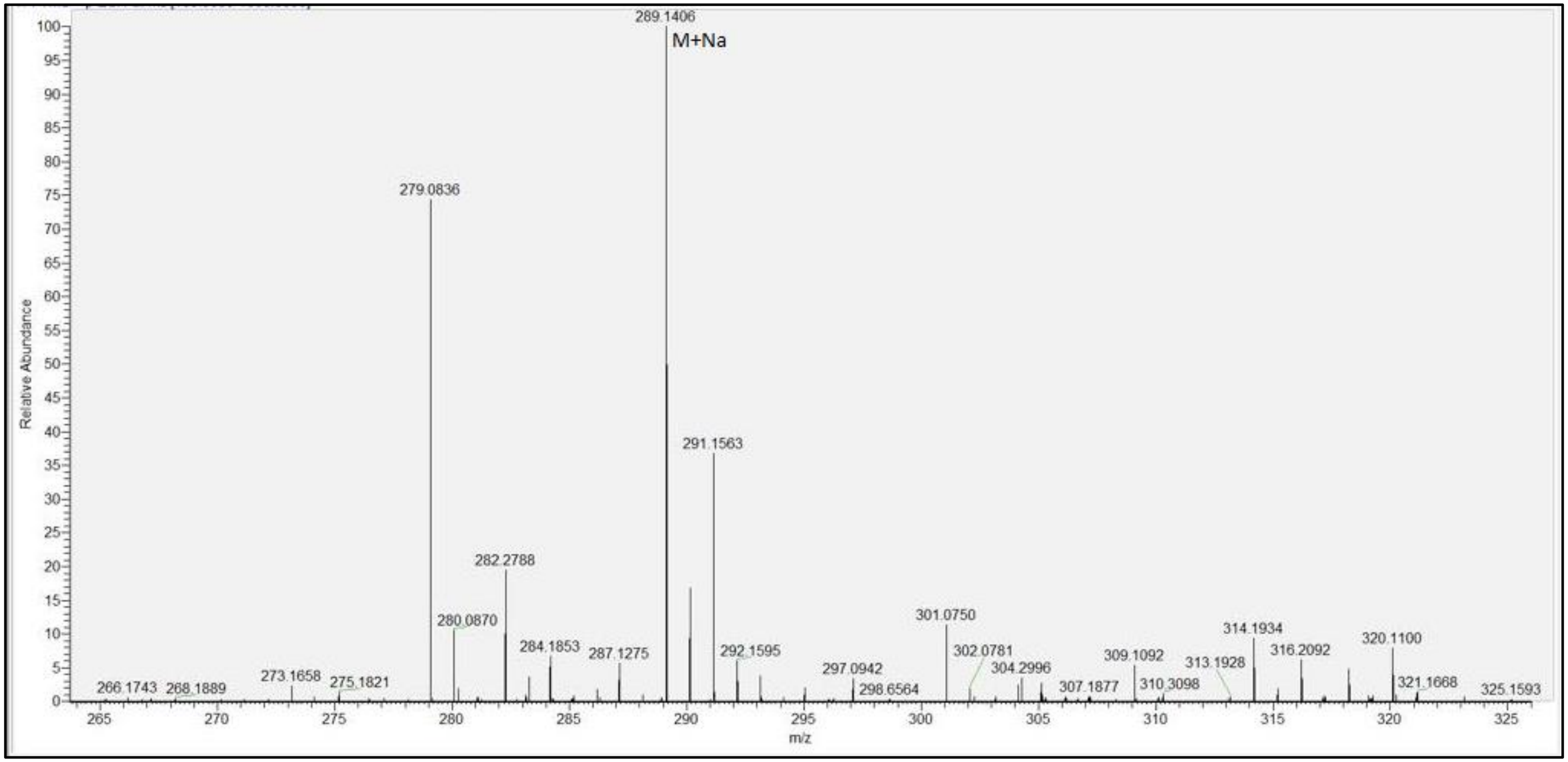

Figure S12: HRESIMS spectrum of **(3a)**

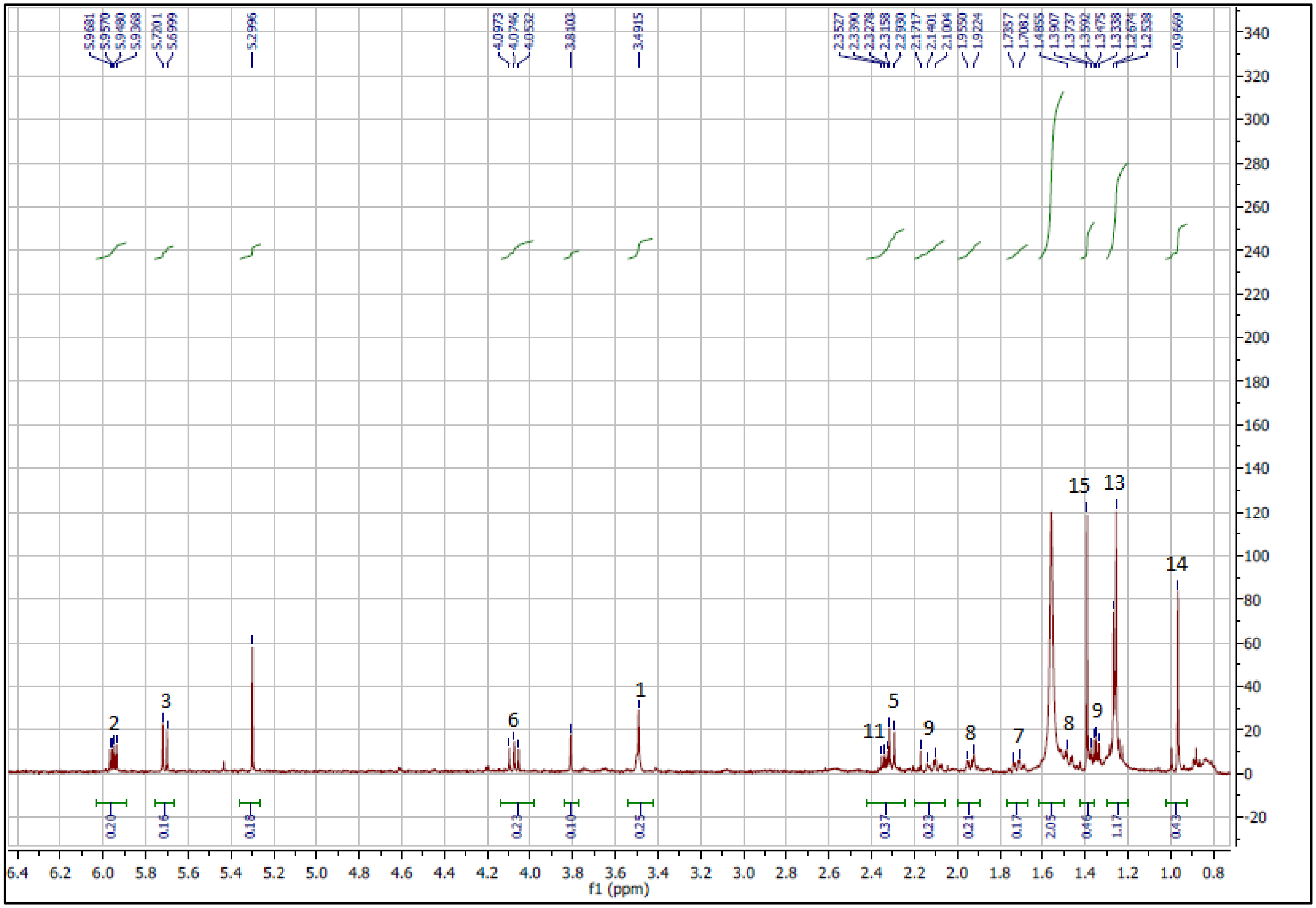

Figure S13: ^{1}H-NMR spectrum of **(3a)** ($CDCl_3$, 500MHz).

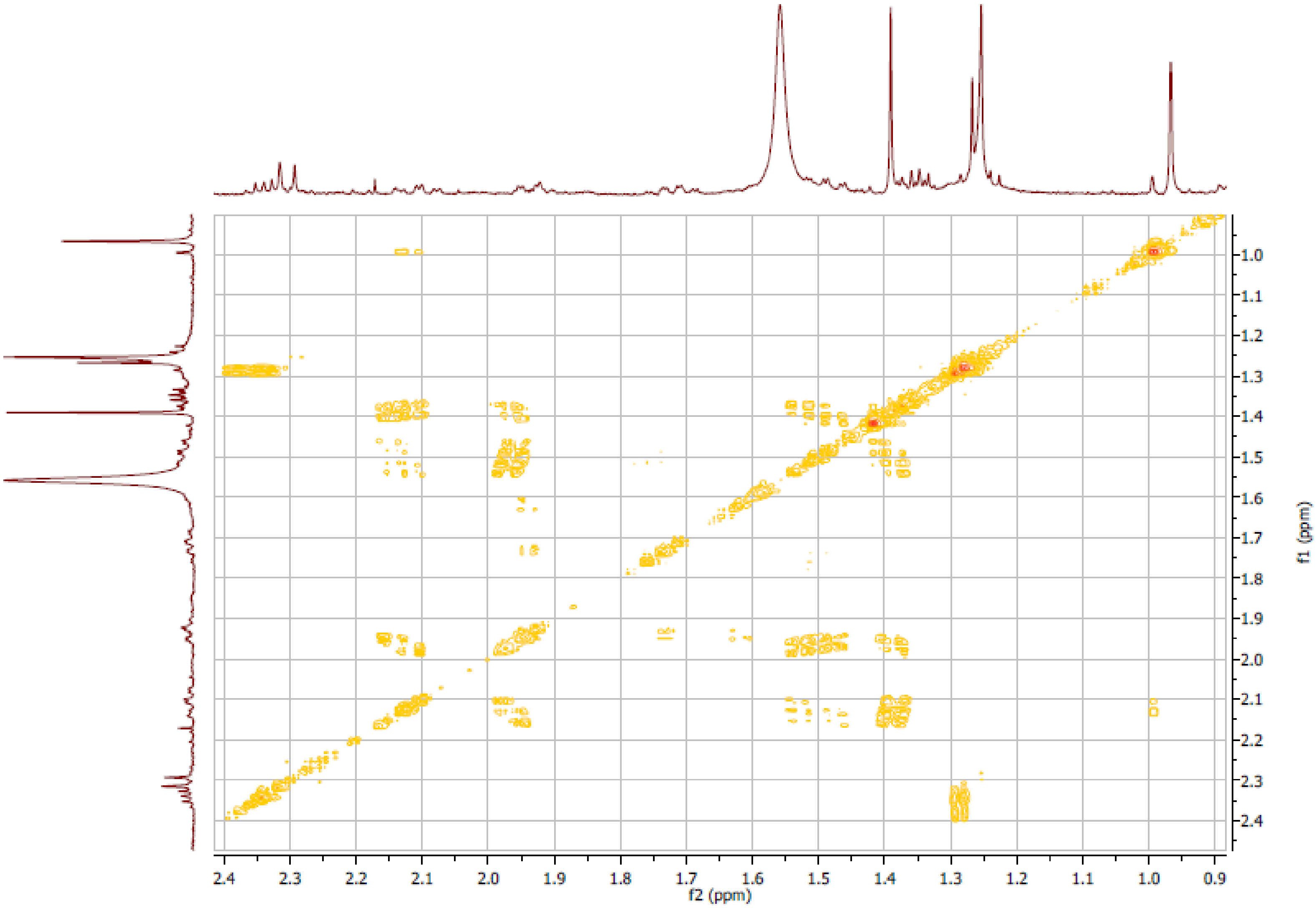

Figure S14: COSY spectrum of **(3a)**

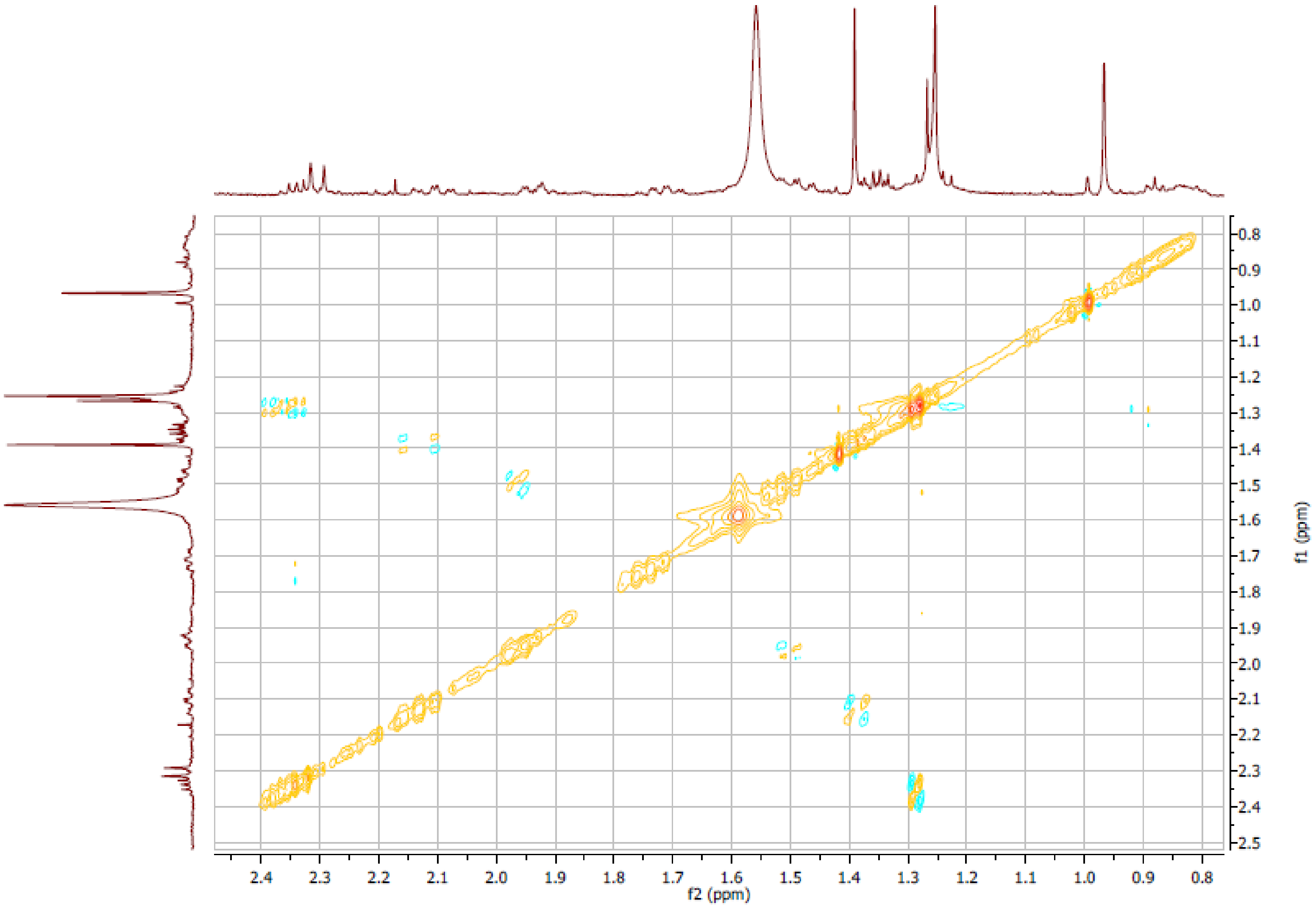

Figure S15: NOESY spectrum of **(3a)**

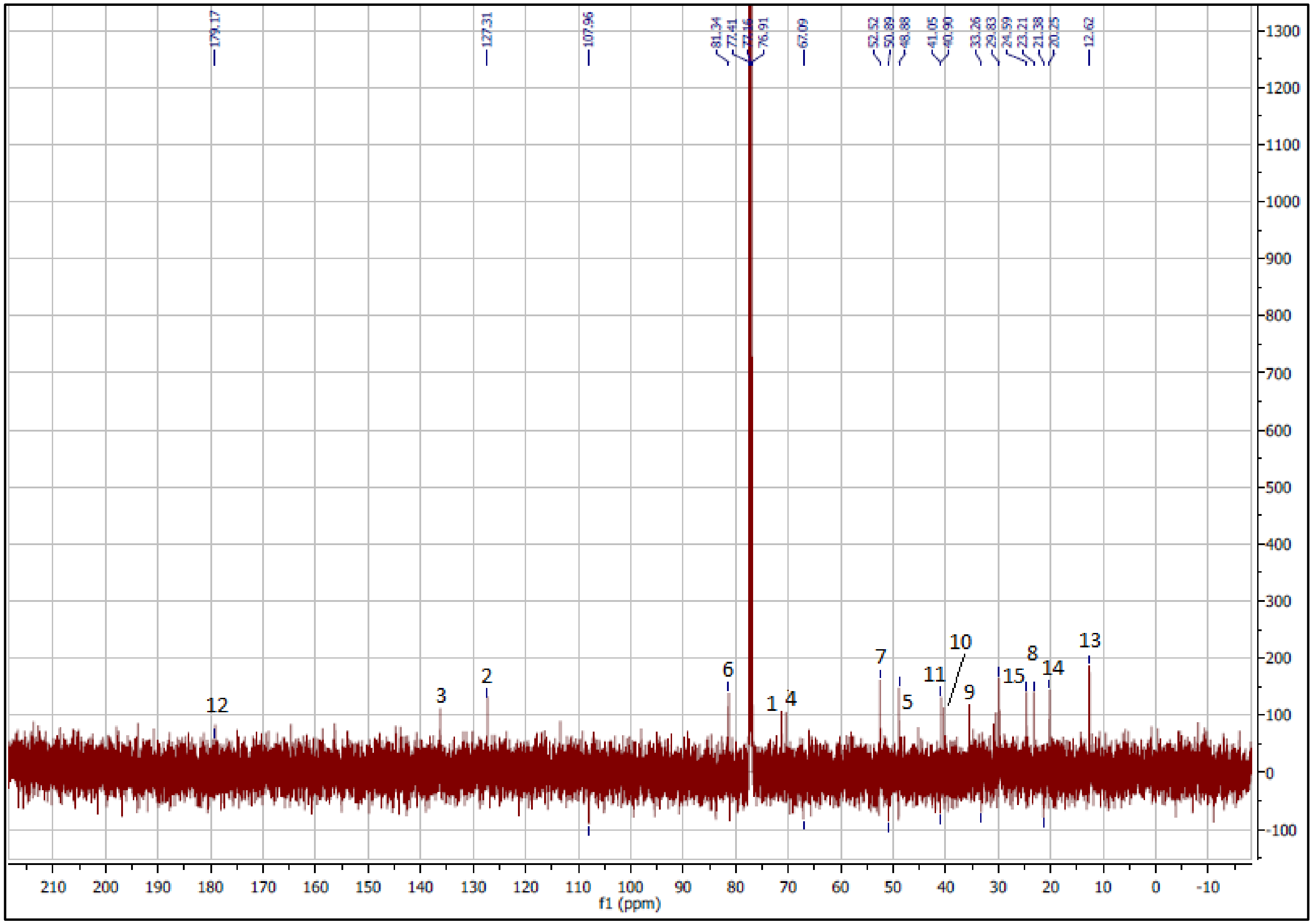

Figure S16: ^{13}C-NMR spectrum of **(3a)** ($CDCl_3$, 125 MHz).

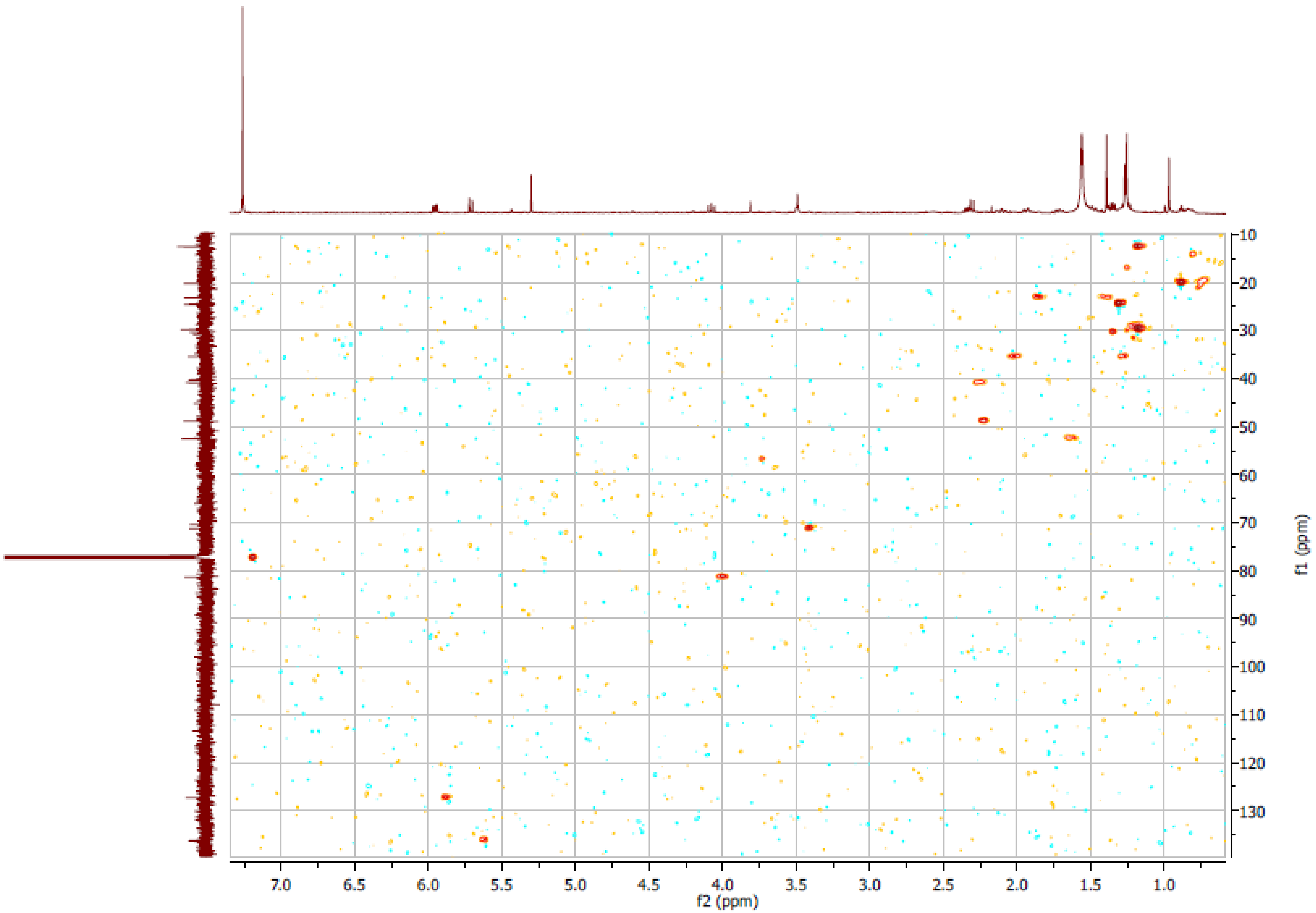

Figure S17: HSQC spectrum of **(3a)**

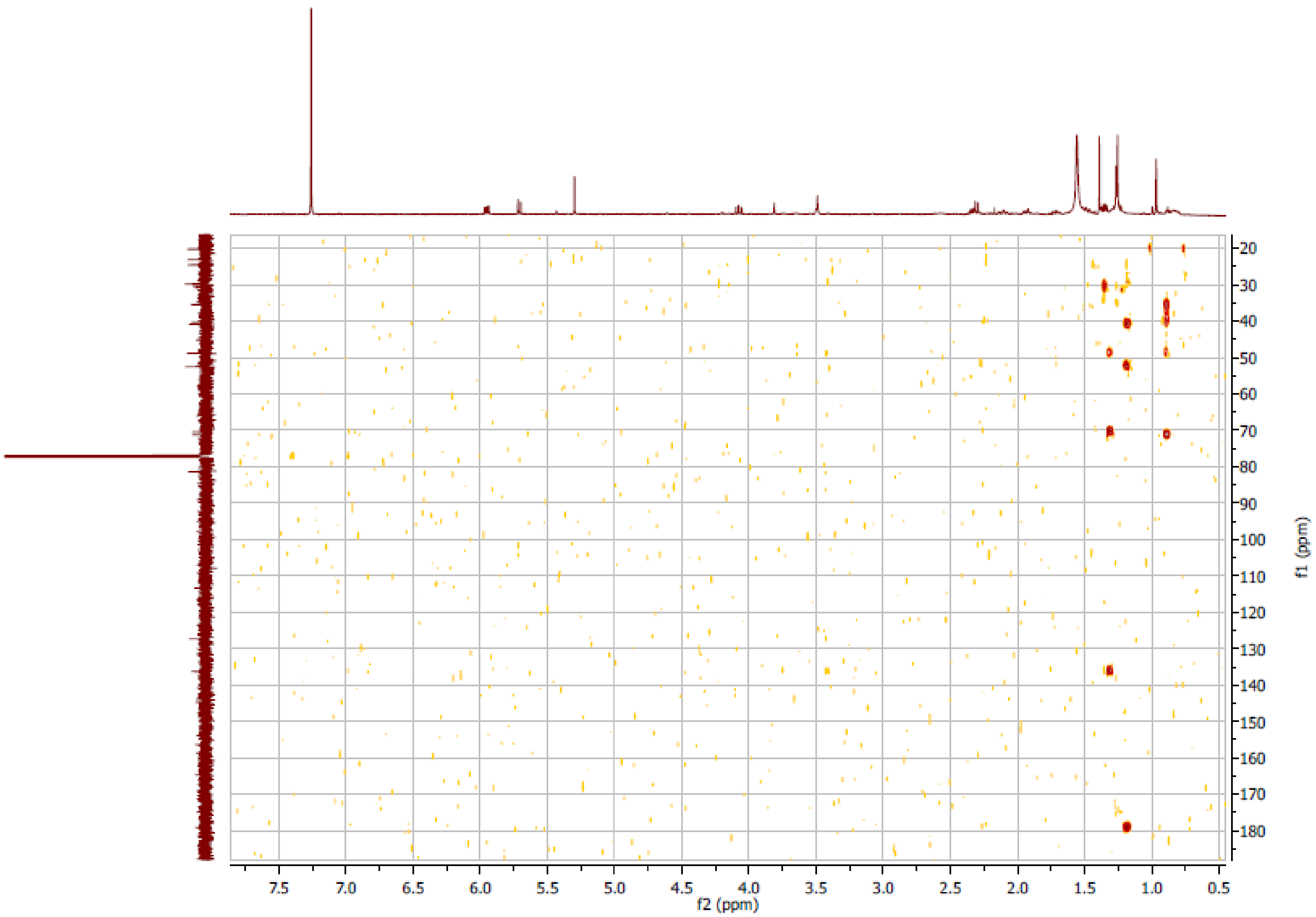

Figure S18: HMBC spectrum of **(3a)**

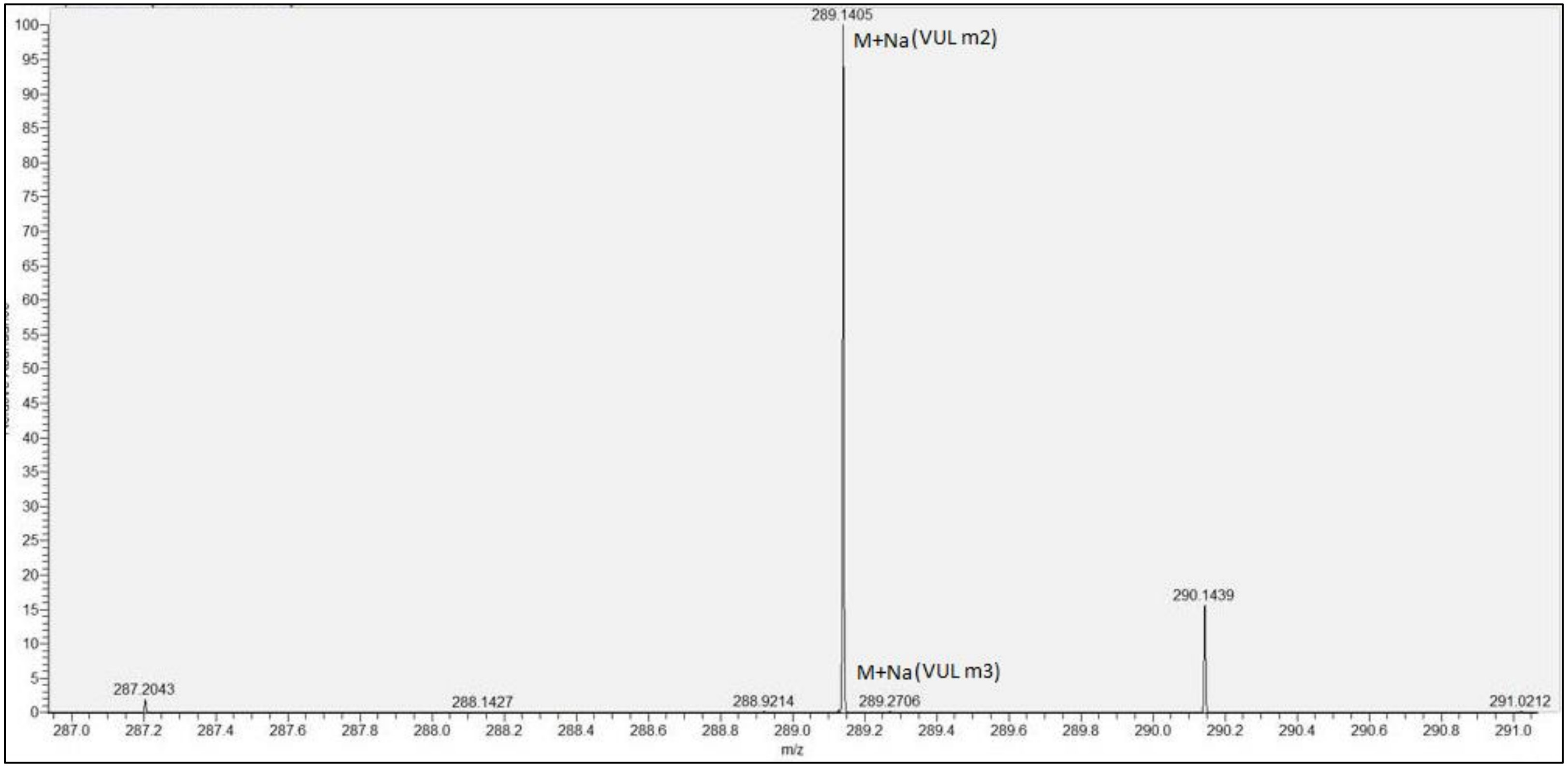

Figure S19: HRESIMS spectrum of **(3a, b)**

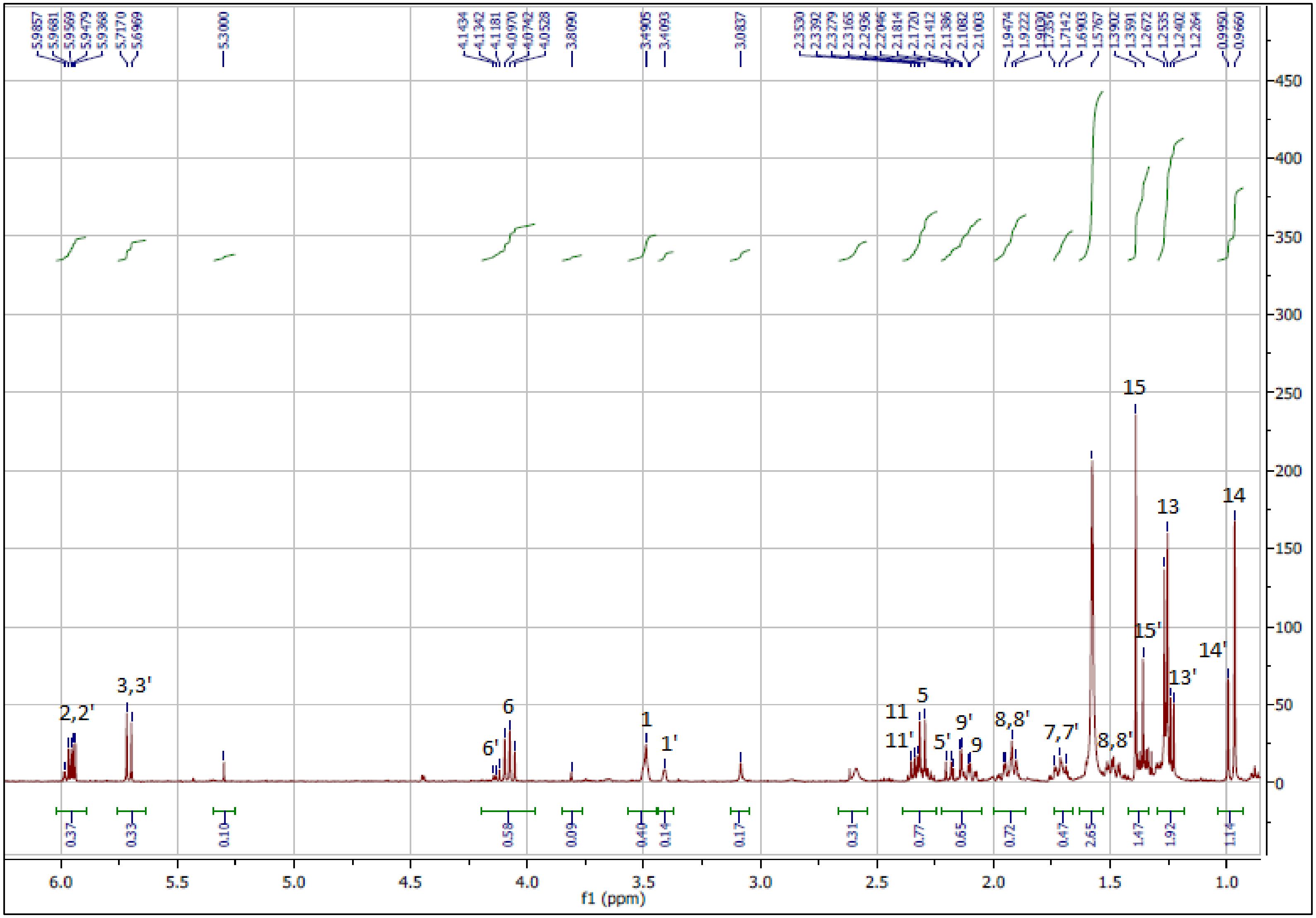

Figure S20: ^{1}H-NMR spectrum of **(3a,b)** ($CDCl_3$, 500 MHz).

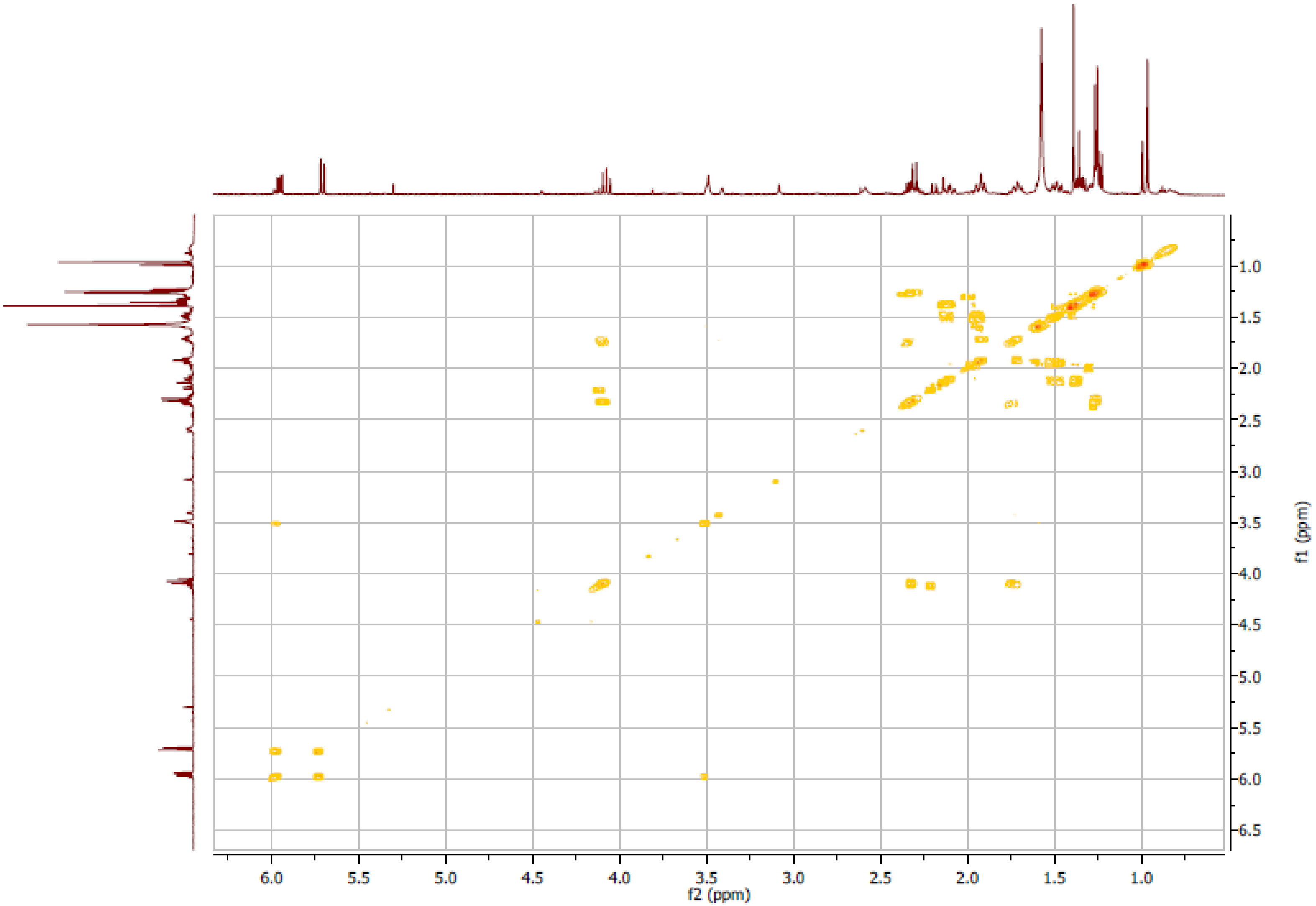

Figure S21: COSY spectrum of **(3a,b)**

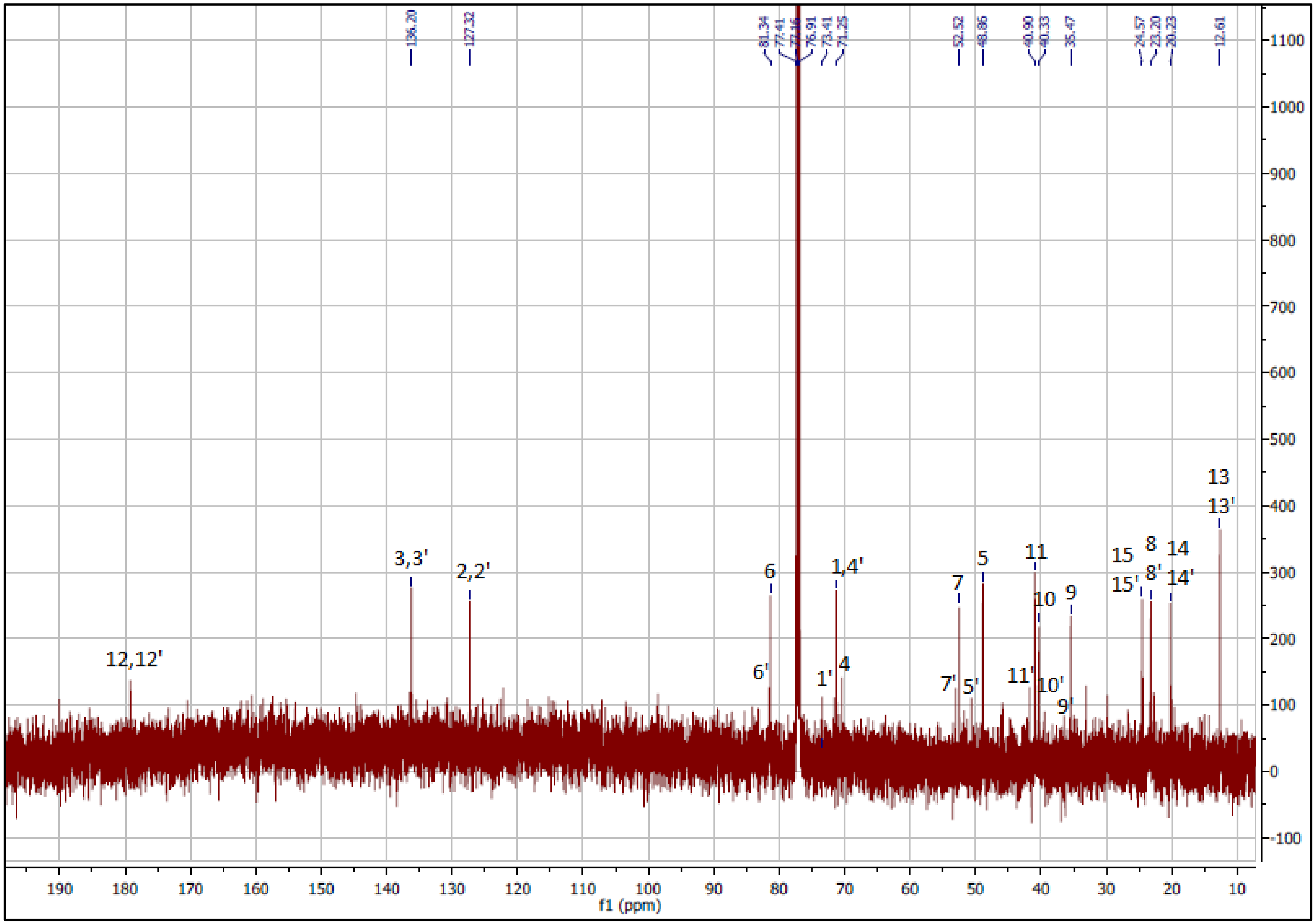

Figure S22: ^{13}C-NMR spectrum of **(3a,b)** ($CDCl_3$, 125 MHz).

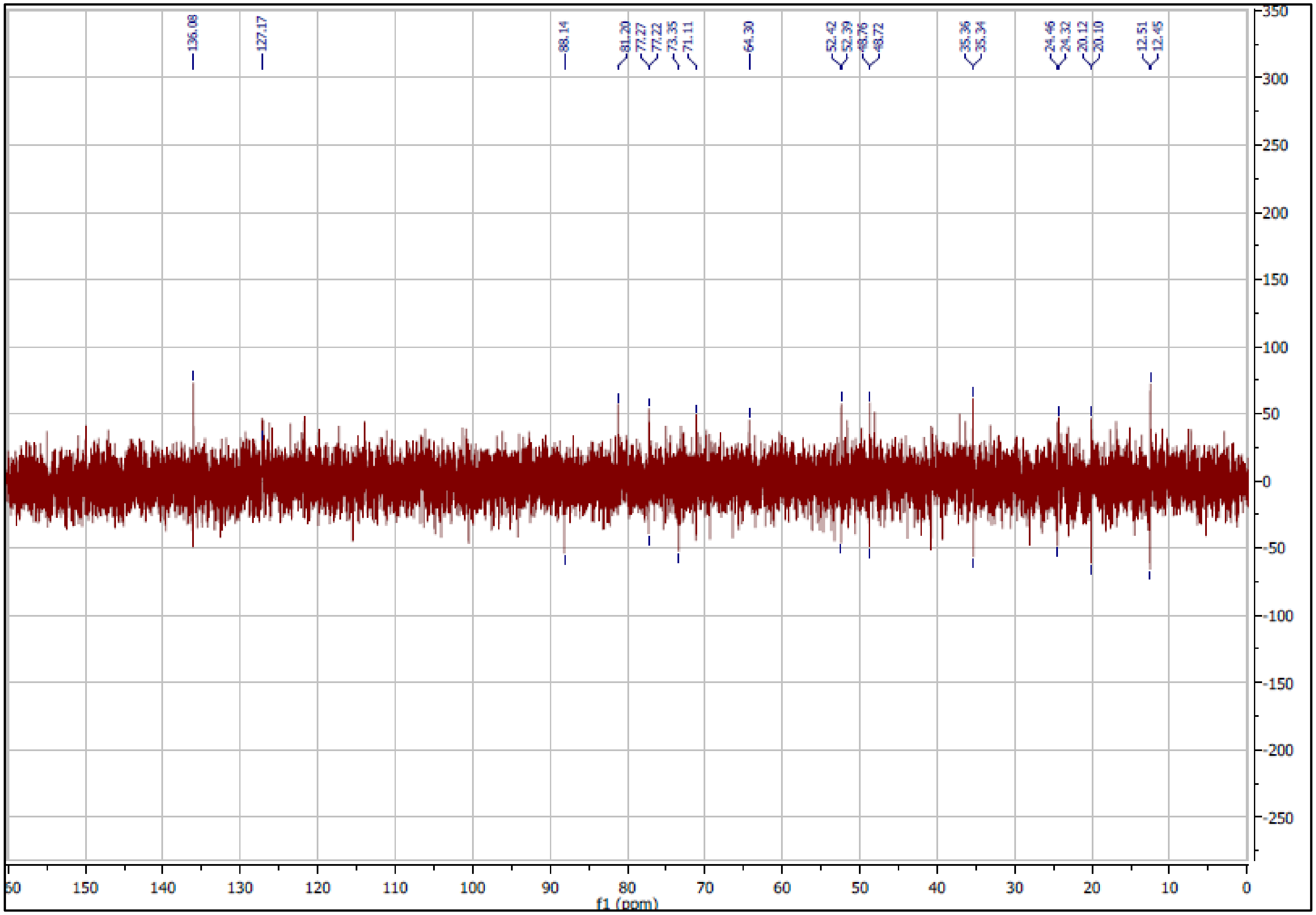

Figure S23: DEPT 135 spectrum of **(3a,b)**

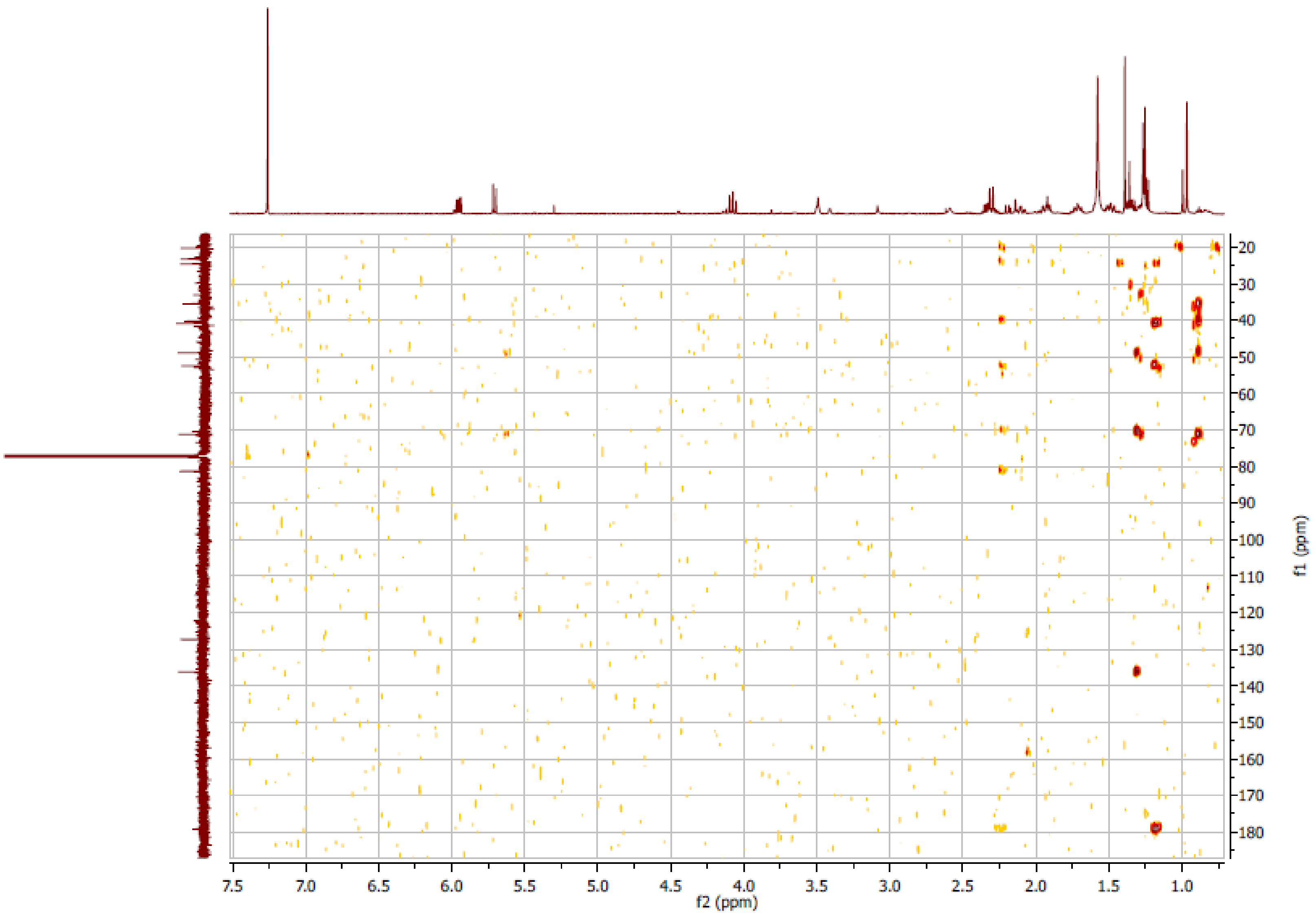

Figure S24: HMBC spectrum of **(3a,b)**

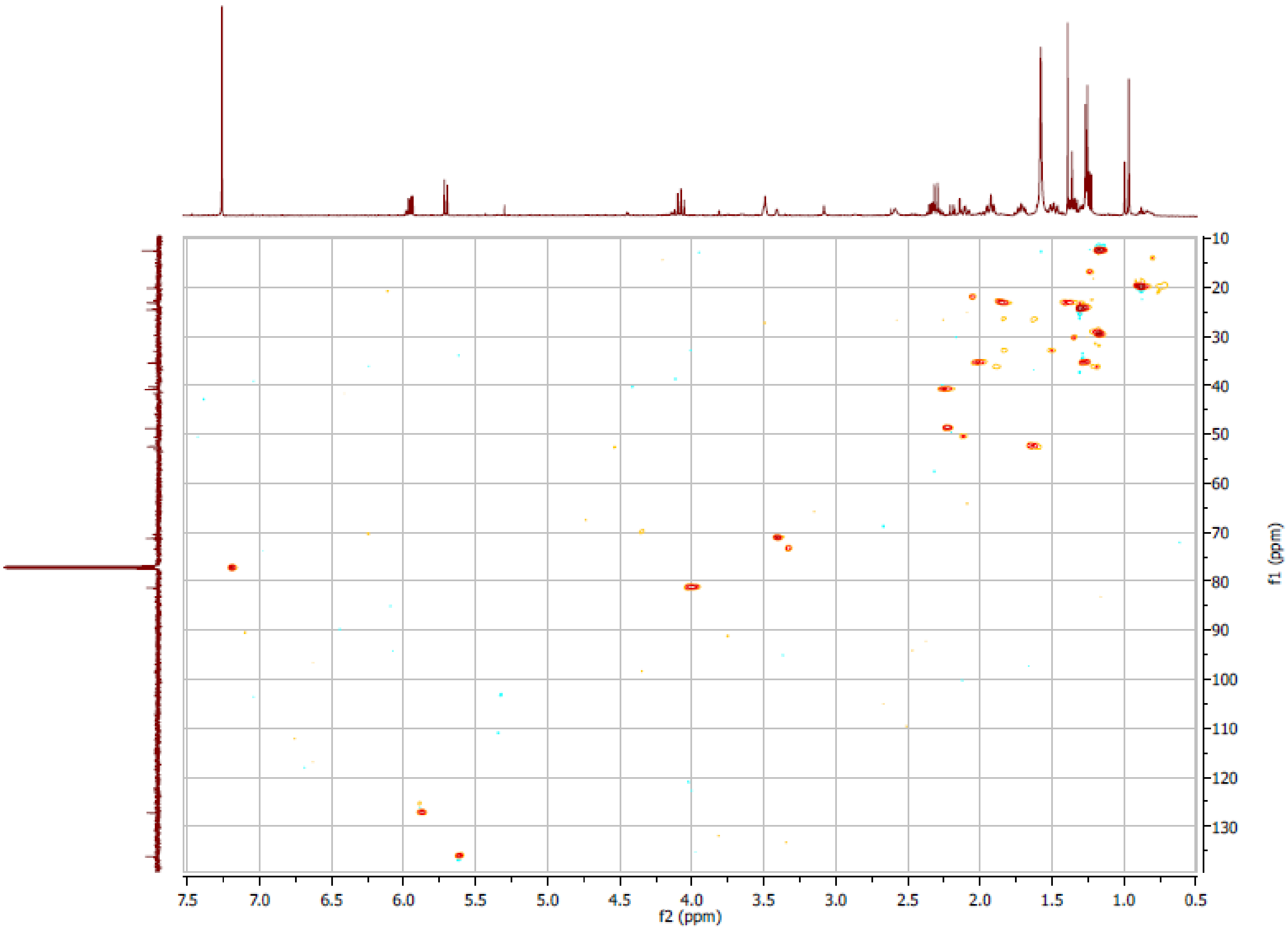

Figure S25: HSQC spectrum of **(3a,b)**
